# Exploring the Appropriate Price of Semaglutide for Type 2 Diabetes Patients Based on Cost-Utility Analysis in China

**DOI:** 10.3389/fphar.2021.701446

**Published:** 2021-06-10

**Authors:** Shanshan Hu, Xiaorong Su, Xun Deng, Yong Wang

**Affiliations:** ^1^ Department of Pharmacy, Zhujiang Hospital, Southern Medical University, Guangzhou, China; ^2^ Department of Clinical Pharmacy, Shanghai General Hospital, Shanghai Jiao Tong University School of Medicine, Shanghai, China; ^3^ Laboratory of Research of New Chinese Medicine, Zhujiang Hospital, Southern Medical University, Guangzhou, China

**Keywords:** cost-utility analysis, type 2 diabetes, UKPDS outcome model, semaglutide, empagliflozin, price exploring

## Abstract

**Introduction:** Semaglutide is the first and only oral version of a glucagon-like peptide-1 analogue approved by the FDA for the treatment of type 2 diabetes (T2D). This research was designed to explore the appropriate price of once-weekly (OW) semaglutide for T2D patients in China based on cost-utility analysis.

**Methods:** The baseline patient cohorts of OW semaglutide and once-daily (OD) empagliflozin were sourced from a patient-level meta-analysis integrating the SUSTAIN 2, SUSTAIN 3, SUSTAIN 8 and PIONEER 2 trials. The long-term health and economic outcomes were simulated using the United Kingdom Prospective Diabetes Study Outcome Model 2 from the Chinese healthcare provider’s perspective. The appropriate price of semaglutide was explored by binary search. One-way sensitivity analysis (one-way SA), probabilistic sensitivity analysis and scenario analysis were applied to solve the uncertainty.

**Results:** Under the assumption that the annual cost of semaglutide is equal to that of OD empagliflozin, OW semaglutide was superior to OD empagliflozin due to its higher quality adjusted life years and lower total costs. After binary search, the incremental cost-utility ratio of OW semaglutide vs. OD empagliflozin was approximately equal to 3*λ* with an annual cost of semaglutide of $1,007.18 and approximately equal to *λ* with an annual cost of semaglutide of $708.11. Subsequently, the incremental cost-utility ratio of OW semaglutide vs. OD empagliflozin was approximately 3*λ* and *λ*, with annual costs of semaglutide of $877.43 and $667.04, respectively, adjusted by one-way SA. Ultimately, the cost-utility results with annual costs of semaglutide of $877.43 and $667.04 were robust to probabilistic sensitivity analysis and scenario analysis.

**Conclusion:** In conclusion, the annual cost of semaglutide appears to be appropriate between $667.04 and $877.43 for T2D patients in China.

## Introduction

Diabetes mellitus is a serious and growing public health challenge in the 21st century, that imposes a tremendous economic burden on national healthcare. It was estimated that in 2019, there were approximately 116.4 million adults with diabetes (20–79 years of age) in China, which is currently the country with the highest number of diabetic patients in the world (International Diabetes Federation, 2019). The latest epidemiological study showed that the prevalence of diabetes is approximately 11%, and type 1 diabetes accounts for fewer than 5% of diabetes cases in China (Ma, 2018). Moreover, a previously published report showed that China had 823,800 deaths from diabetes in 2019 (International Diabetes Federation, 2019). The expenditures caused by diabetes exerts a significant impact on health budgets in China, as it was estimated that diabetes-related health expenditures totaled approximately USD 109.0 billion in China in 2019 (International Diabetes Federation, 2019). Furthermore, previous studies demonstrated that the direct medical costs of diabetes patients with complications are significantly higher than those of uncomplicated patients (Wang et al., 2009; Moucheraud et al., 2019). It is therefore pressing that cost-effective therapies for managing diabetes are developed to reduce the health and economic burden.

Diabetes is a chronic condition requiring lifelong management. Intensive self-management is essential for patients with diabetes to achieve good metabolic control (Chatterjee et al., 2017). Currently, metformin is the first-line treatment for patients with type 2 diabetes (T2D) worldwide. Guidelines published by the American Diabetes Association (ADA) and the European Society for the Study of Diabetes (EASD) recommend either glucagon-like peptide 1 (GLP-1) receptor agonists or sodium-glucose cotransporter 2 (SGLT2) inhibitors as second-line therapies for patients with T2D on metformin monotherapy or as first-line treatments for patients with T2D at high-risk of cardiovascular events or seeking to minimize weight gain (Davies et al., 2018; Marx et al., 2021; Cosentino et al., 2020). Moreover, GLP-1 receptor agonists (GLP-1 RAs) and SGLT2 inhibitors can also reduce the risk of hypertensive heart failure, cardiovascular death, and chronic kidney disease (Marx et al., 2021).

Semaglutide, a newly approved GLP-1 RA, promotes insulin secretion and inhibits the secretion of glucagon through a glucose concentration-dependent mechanism. Patients with T2D have greatly improved blood glucose levels and a lower risk of hypoglycemia (Drucker, 2018; Maruthur et al., 2016; Andersen et al., 2018). Empagliflozin is an inhibitor of SGLT-2, and has been proven to block the reabsorption of glucose in the kidney and excrete excess glucose into the body, thereby lowering blood glucose levels (Heerspink et al., 2016; Zheng et al., 2018; Rodbard et al., 2019; Strøm Halden et al., 2019). Despite the divergent mechanisms of semaglutide and empagliflozin, their functions and curative effects are remarkably similar. The leading position of semaglutide that is mainly reflected in the data results from a series of clinical projects, named SUSTAIN one to seven, that were published by Novo Nordisk (Sorli et al., 2017; Ahrén et al., 2017; Ahmann et al., 2018; Aroda et al., 2017; Rodbard et al., 2018; Pratley et al., 2018; Marso et al., 2016). After a head-to-head clinical trial comparison with other hypoglycemic drugs, semaglutide showed great advantages in hypoglycemia, weight loss and safety. At the same time, semaglutide is also the third hypoglycemic agent to show cardiovascular benefits after empagliflozin and liraglutide and can reduce the incidence of cardiovascular events by 26% (Marso et al., 2016). Furthermore, semaglutide is the first and only FDA-approved oral version of a GLP-1 receptor agonist. China, as the country with the largest number of diabetes patients, urgently needs a cost-effective diabetes treatment plan.

Recently, a considerable amount of literature has grown around the theme of burden in T2D, as T2D is chronic and progressive. Various hypoglycemic drugs are swarming into the Chinese market, and semaglutide is one of them. To date, there has been no pharmacoeconomic assessment of semaglutide vs. empagliflozin for T2D patients in China. The aim of this study is to explore the appropriate price of once-weekly semaglutide for T2D patients based on cost-utility analysis in China including once-daily empagliflozin 25 mg as a comparator.

## Methods

### Model Overview

A cost-utility analysis was performed by using version 2.0 of the United Kingdom Prospective Diabetes Study Outcome Model (UKPDS OM2). The UKPDS OM2, a computerized simulation model that has been validated and is widely used by researchers (Pollock et al., 2019), was developed to assess the long-term health and economic outcomes of interventions for T2D. The detailed structure and algorithms of the model were described in our previous paper (Hu et al., 2020). For simplicity, the long-term health and economic outcomes of T2D can be simulated by using the risk equations from the UKPDS 82 (Hayes et al., 2013). The model inputs include demographic characteristics, risk factor values, pre-existing events, specified costs, health utility and other relevant parameters. The model outputs include life expectancy (LE), quality-adjusted life years (QALYs), therapy cost, complication cost and total cost. Mean values and 95% confidence intervals (CIs) were output for long-term health and economic outcomes. The UKPDS OM2 contains a series of semi-Markov structures with default annual cycles over a user-defined time horizon within 70 years. In this study, the mean age of the patient cohort was 56 years. Therefore, the time horizon was preset to 40 years to capture all related long-term complications and associated costs in the patient’s lifetime. The annual discount rate was preset at 5% (Hou et al., 2019) for both future costs and utilities in line with WHO guidelines (Organization, 2003). Moreover, a second-order Monte Carlo simulation (Gao et al., 2012), which sampled baseline characteristics, clinical efficacy, costs and utilities related to the patient cohort, was performed by the UKPDS OM2 to solve parameter uncertainties. This research was based on previous studies and did not involve human participants or animals.

### Baseline Cohort Characteristics and Clinical Efficacy

To date, no head-to-head randomized clinical trial has been conducted on 1 mg once-weekly (OW) semaglutide compared with 25 mg once-daily (OD) empagliflozin. Therefore, the baseline patient cohort was sourced from a patient-level meta-analysis (Lingvay et al., 2020) that integrated the clinical efficacy data of the SUSTAIN 3 (Ahmann et al., 2018) (OW semaglutide vs. OW exenatide), SUSTAIN 2 (Ahrén et al., 2017) (OW semaglutide vs. OD sitagliptin), SUSTAIN 8 (Lingvay et al., 2019) (OW semaglutide vs. OD canagliflozin) and PIONEER 2 (Rodbard et al., 2019) (OD semaglutide vs. OD empagliflozin) trials. In this study, the total simulation sample was assumed to be 1,000 in the OW semaglutide arm and OD empagliflozin arm. The demographic characteristics of the patient cohort of mean [standard deviation (SD)] age and mean duration of diabetes were 56 (10.3) years and 7 (5.9) years, respectively. The risk factor values of mean initial HbA1c, mean BMI, and high-density lipoprotein (HDL) levels were 8.2 (1.0)%, 32.8 (6.7) and 1.2 (0.3) mmol/L, respectively. The detailed data on pre-existing events and other data are shown in Table 1. The efficacy results (e.g., ΔHbA1c, ΔBMI…) were extracted from the patient-level meta-analysis (Lingvay et al., 2020) and are listed in Table 2. The meta-analysis reported that the OW semaglutide significantly decreased mean HbA1c vs. OD empaglifiozin, by 1.44 vs. 0.83% (*p* < 0.0001), respectively. Unreported data were substituted by the model defaults. Moreover, T2D is a chronic disease with progressive damage to beta-cell function, which results in most T2D patients eventually requiring insulin administration (Gao et al., 2012). Hence, to simulate clinical practice, the treatment time was preset to 5 years (Gao et al., 2012). Treatment with basal insulin was assumed to initiate when the treatment time finished. Usually, basal insulin is assumed to be generic insulin glargine.

**TABLE 1:** Baseline characteristics of simulation cohort.

Trial characteristics	Mean	SD
Total simulation sample	1,000	
Mean age, years	56	10.3
Female, %	48	
Race, %		
White	77	
Black/African American	6.84	
Asian	14	
American Indian/Alaskan Native	0.25	
Other^*^	1.91	
Mean duration of diabetes, year	7	5.9
Mean HbA1c, %	8.2	1.0
Mean BMI, kg/m^2^	32.8	6.7
SBP, mmHg	131.41	14.6
DBP, mmHg	80.01	9.5
TC, mmol/L	4.75	1.09
HDL cholesterol, mmol/L	1.20	0.30
LDL cholesterol, mmol/L	2.64	0.88
Triglycerides, mmol/L	2.12	1.55
eGFR, ml/min/1.73 m^2^	97.35	15.34
History Of MI	4%	
History Of angina	2%	
History Of PVD	1%	
History Of renal complications	Few	
History Of microalbuminuria	Less 1%	
History Of background diabetic retinopathy	8%	
Smoking status, %		
Current	14.22	
Previous/never	85.78	

MI: myocardial infarction; PVD: peripheral vascular disease.

Data source: Capehorn et al., 2021; Lingvay et al., 2020

^*^Includes patients whose race was not available in study records.

**TABLE 2:** Mean changes from baseline at 52 weeks efficacy end points.

Parameters	Arm SEMA, *n* = 1,000		Arm EMPA, *n* = 1,000		Estimated treatment difference (95% CI)	*p* value*
	Mean	SD	Mean	SD		
HbA1c, %	−1.44	0.03	−0.83	0.05	−0.61 [−0.72 to −0.49]	<0.0001
SBP, mmHg	−4.11	0.36	−4.48	0.56	0.37 [−0.95 to 1.68]	0.5842
DBP, mmHg	−1.27	0.23	−2.39	0.37	1.12 [0.27 to 1.97]	0.0103
TC, mmol/L	−6.15	0.90	4.14	1.39	−10.28 [−13.56 to −7.01]	<0.0001
HDL cholesterol, mmol/L	1.53	0.22	2.63	0.34	−1.10 [−1.89 to −0.30]	0.0073
LDL cholesterol, mmol/L	−2.48	0.77	4.18	1.19	−6.66 [−9.44 to −3.87]	<0.0001
Triglycerides, mmol/L	−31.16	3.36	−15.13	5.17	−16.03 [−28.17 to −3.90]	0.0097
BMI, kg/m^2^	−1.92	0.06	−1.32	0.09	−0.60 [−0.81 to −0.39]	<0.0001
eGFR, ml/min/1.73 m^2^	0.15	0.23	−0.06	0.37	0.21 [−0.65 to 1.07]	0.6304
Waist circumference, cm	−4.66	–	−2.76	–	−1.90 [−2.54 to −1.26]	<0.0001

SBP: Systolic blood pressure; DBP: Diastolic blood pressure; TC: Total cholesterol; eGFR: Estimated glomerular filtration rate.

–, Not report.

Data source: Capehorn et al., 2021; Lingvay et al., 2020

*All signifcant p values (p < 0.05) favor SEMA, except DBP and HDL cholesterol, which favor EMPA.

### Costs and Utilities

The direct medical costs, including medication costs, diabetes management costs and costs of complications related to T2D, were taken into account from the Chinese healthcare provider’s perspective in 2019 US dollars. The annual costs of 1 mg OW semaglutide and 25 mg OD empagliflozin were captured as medication costs. Nevertheless, semaglutide is the first and only FDA-approved oral version of a GLP-1 receptor agonist. Therefore, the price of OW semaglutide could not be found because OW semaglutide has not been listed on the Chinese stock market. The annual cost of OW semaglutide was assumed to be equal to the annual cost of OD empagliflozin at the beginning of the study. The price of OD empagliflozin was sourced from the out-of-pocket (OOP) price in 2020. After a calculation, the annual cost of OD empagliflozin was $558.2. The diabetes management costs and costs of complications related to T2D were derived from previous literature with respect to a Chinese economic evaluation (Li et al., 2019; Shao et al., 2017; Hou et al., 2019; Cai et al., 2019). Data on health state utility and disutility scores and the initial utility score without complications were extracted from a 5-level, 5-dimensional EuroQol scale (EQ–5D–5L) study with T2D patients in China (Pan et al., 2016). Other utility data not mentioned in the EQ–5D–5L study were supplemented from the UKPDS 62 study (Clarke et al., 2002). Detailed data about costs and utilities are shown in Table 3. All the costs were expressed as 2019 United States dollars (1 United States dollar = 6.8 Chinese Yuan).

**TABLE 3:** Key model inputs of costs and utilities.

Complications	At time of event			In subsequent years	
	Fatal cost	Non-fatal cost	Utility decrement	Cost	Utility decrement
IHD	0.00^a^	6,293.30^a^	−0.090^b^	1,123.51^a^	−0.090^b^
MI	7,855.14^a^	7,855.14^a^	−0.055^b^	484.52^a^	−0.236^a^
Heart failure	3,033.73^b^	3,033.73^b^	−0.236^a^	1,604.12^a^	−0.236^a^
Stroke	2,266.31^b^	3,059.07^a^	−0.164^b^	539.32^a^	−0.326^a^
Amputation	4,434.60^a^	4,434.60^a^	−0.380^a^	4,316.66^a^	−0.380^a^
Blindness	–	2,361.49^b^	−0.157^a^	1,747.01^a^	−0.157^a^
Renal failure	0.00^a^	14,685.91^a^	−0.400^a^	14,685.91^a^	−0.400^a^
Ulcer	–	2,310.00^b^	−0.059^b^	813.01^b^	−0.059^b^
Initial utility	0.876^a^ ^,^ ^c^				
Cost in the absence of complications	1,427.10^d^				

a Data sourced from Hou et al. (2019).

b Data sourced from Cai et al. (2019).

c Data sourced from Pan et al. (2016).

d Data sourced from Li et al. (2019).

### Cost-Utility Analysis

Cost-utility analysis was evaluated by the QALYs and total cost in each group output by the UKPDS OM2 and the incremental cost-utility ratio (ICUR) was calculated with the OW semaglutide group vs. the OW exenatide group. The willingness-to-pay (WTP) threshold referred to 1–3 times the GDP per capita as recommended by the WHO. In this research, the value of GDP was deemed *λ* and that of 3 times the GDP was deemed 3*λ*. The WTP threshold was set at *λ* for the “very cost effective” threshold and set at 3*λ* for the “cost effective” threshold. The relationship between the ICUR and WTP threshold is shown as following:

If ICUR < *λ*, then indicating that the incremental cost of OW semaglutide group vs. OD empagliflozin group appears to be definitely worthwhile, which means OW semaglutide group was a very cost-effective therapy.
If *λ* < ICUR < 3*λ*, then the incremental cost of OW semaglutide vs. OD empagliflozin appears to be acceptable, which means that OW semaglutide was a cost effective therapy.
If ICUR > 3*λ*, then the incremental cost of OW semaglutide vs. OD empagliflozin does not appear to be worthwhile.

### Sensitivity Analysis

One-way sensitivity analysis (one-way SA), probabilistic sensitivity analysis (PSA) and scenario analysis were performed to measure the robustness of the base assumption.

In one-way SA, the parameters included costs of complications, health disutility scores, initial utility score, treatment time, time horizon and discount rate. Simulations were run with time horizons of 30 and 50 years and with treatment times of 4 and 6 years. Simulations were operated with an initial utility of 0.78 and 0.92 and with discount rates of 3% and 8%. Costs of complications and health disutility scores varied between their 95% CIs. Costs and utility scores were adjusted by ± 20% and ± 10%, respectively, if data on 95% CIs were not reported. The detailed parameters for one-way SA are depicted in Table 4. The results of one-way SA are reported as tornado diagrams.

**TABLE 4:** Parameters for sensitivity analysis.

No.	Parameters	Baseline	Low	High
1	Discount rate	5%	3%	8%
2	Initial utility	0.876	0.78	0.92
3	Treatment time, years	5	4	6
4	Time horizon, years	40	30	50
*Cost, $*				
5	IHD per year cost (±20%)^*^	1,123.51	898.808	1,348.212
6	MI per year cost	484.52	307.06	661.99
7	CHF per year cost	1,604.12	1,334.83	2,800.64
8	Stroke per year cost	539.32	474.42	880.95
9	Blindness per year cost	1747.01	1,521.87	1972.03
10	ERSD per year cost	14,685.91	13,994.99	15,500.9
11	Amputation per year cost	4,316.64	0	7,669.32
12	Ulcer per year cost (±20%)^*^	813.01	650.408	975.612
*Health disutility scores*				
13	IHD disutility scores (±10%)^*^	0.09	0.081	0.099
14	MI disutility scores	0.236	0.026	0.446
15	CHF disutility scores	0.236	0.026	0.446
16	Stroke disutility scores	0.326	0.036	0.616
17	Blindness disutility scores	0.157	0.007	0.307
18	ERSD disutility scores	0.4	0.19	0.61
19	Amputation disutility scores	0.38	0.204	0.496
20	Ulcer disutility scores (±10%)^*^	0.059	0.0531	0.0649

* The range data of IHD per year cost, ulcer per year cost, IHD disutility score and ulcer disutility score were not reported. Therefore, we tested IHD and ulcer per year costs as ±20% and IHD and ulcer utility score as ±10%.

The cost for OD empagliflozin was decreased by 20% for scenario analysis. In the PSA, Monte Carlo simulations were applied over 1,000 iterations with input parameters sampled from a fixed probability distribution to address second-order uncertainty, and the results were interpreted as scatter plots of ICUR.

### Binary Search

**FIGURE 1:**
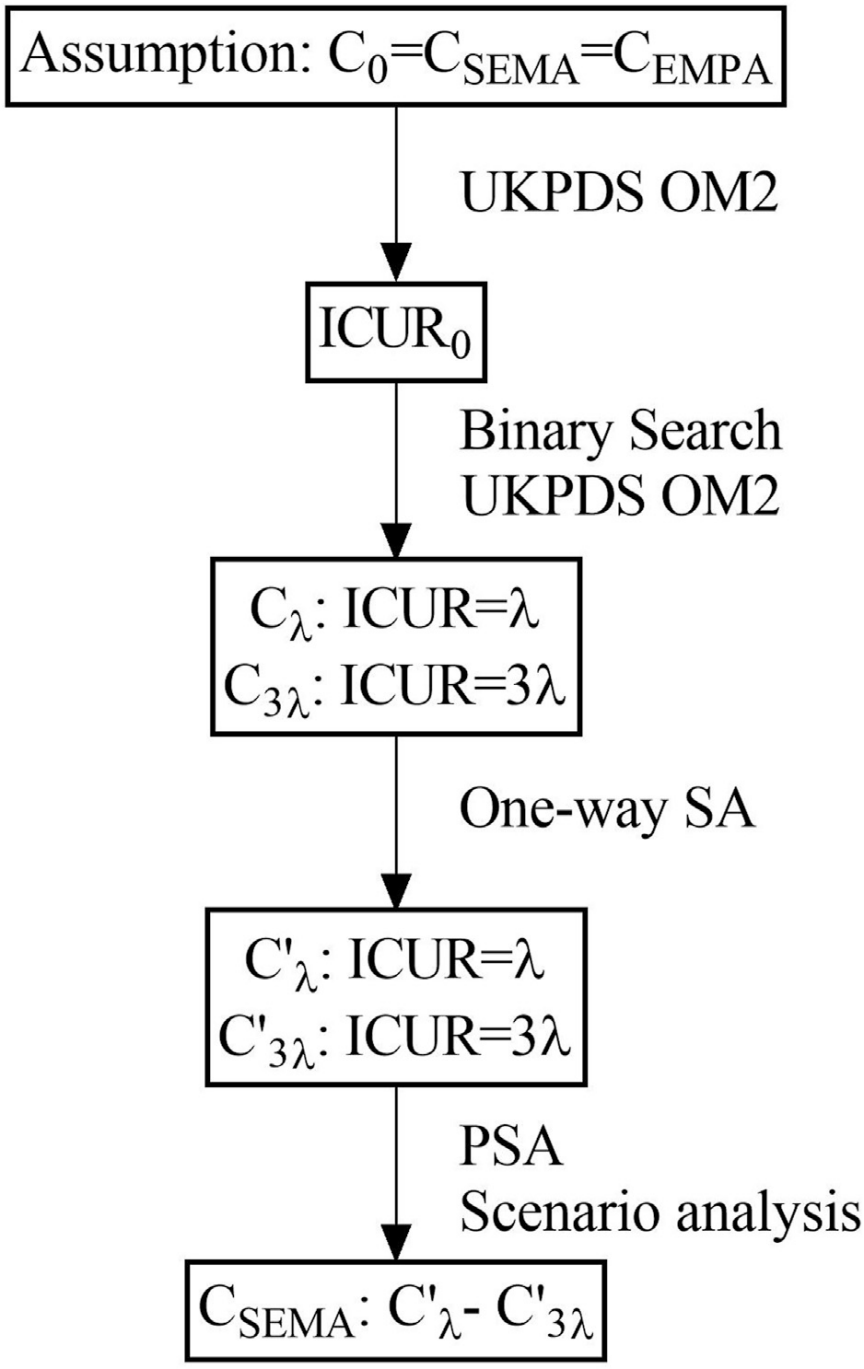
Method flow chart.

Binary search is a fast and effective method to find a specific target value from a set of specific sequences. By starting from the middle of the sequence, it can determine whether it is ascending or descending according to the median compared to the target value, effectively reducing the search space by half. The method flow chat was shown in Figure 1.

## Results

### The Results of Cost-Utility Analysis at the Condition of C_0_ = C_SEMA_ = C_EMPA_

The long-term simulation results for the OW semaglutide group vs. the OD empagliflozin group at the initial assumption of an annual cost of semaglutide equal to that of empagliflozin are shown in Table 5. The QALYs gained in the OW semaglutide group and OD empagliflozin group were 11.11 and 11.01 years, respectively. The total costs were $24358.49 and $24360.95, respectively. Apparently, the OW semaglutide group was superior to the OD empagliflozin group due to its higher QALY and lower total cost.

**TABLE 5:** The results of cost-utility analysis at the condition of C_0_ = C_SEMA_ = C_EMPA_ = $558.2*.

Parameters	SEMA	EMPA	SEMA vs. EMPA
LE, years	13.34	13.26	0.08
QALY, years	11.11	11.01	0.09
Therapy cost, $	3,665.96	3,699.32	−33.36
Cost of complications, $	20,692.53	20,661.62	30.90
Total cost, $	24,358.49	24,360.95	−2.46
ICUR	–	–	Dominance

SEMA: OW semaglutide group; EMPA: OD empagliflozin group; LE: life expectancy; QALY: quality-adjusted life years; ICUR: incremental cost-utility ratio of QALY.

C_0_: initial assumption for annual cost of semaglutide.

C_SEMA_: annual cost of semaglutide.

C_EMPA_: annual cost of empagliflozin.

*Data from www.yaoz.com

### Results for Searching Appropriate Annual Costs for Semaglutide Using Binary Search

As we can see in results 3.1, the OW semaglutide group appears to be dominant over the OD empagliflozin group at the condition of C_0_ = C_SEMA_ = C_EMPA_ = $558.2. Therefore, a series of assumptions were applied to search for appropriate annual costs for semaglutide using binary search. Detailed data on the assumption of annual costs for semaglutide are shown in Supplementary Table S1. The detailed outputs for the cost-utility analysis with assumed annual costs for semaglutide are presented in Supplementary Table S2. When the value of the annual cost for semaglutide was set at $1,007.18, the ICUR of the OW semaglutide group vs. the OD empagliflozin group was $31,275.77, which approached nearly 3*λ* (Table 6). Similarly, when the value of the annual cost for semaglutide was set at $708.11, the ICUR of the OW semaglutide group vs. the OD empagliflozin group was $10,425.24, which was approximately equal to *λ* (Table 6).

**TABLE 6:** Results for searching C_*λ*_ and C_3*λ*_.

C_SEMA_	Group	QALY	∆QALY	Total cost	∆Cost	ICUR	Relationship with *λ*
At C_3λ_ = $1,007.18							
	SEMA	11.1068	0.0942	27,307.12587	2,946.177,869	31,275.77355	≈3*λ*
	EMPA	11.0216		24,360.948			
At C_λ_ = $708.11							
	SEMA	11.1068	0.0942	25,343.0057	982.0576,956	10,425.24093	≈*λ*
	EMPA	11.0216		24,360.948			

SEMA: semaglutide group; EMPA: empagliflozin; LE: life expectancy; QALY: quality-adjusted life years; ICUR: incremental cost-utility ratio of QALY.

C_3λ_: annual cost of semaglutide when ICUR was nearly approach to 3λ.

C_λ_: annual cost of semaglutide when ICUR was nearly approach to *λ*

The value of *λ* was set to GDP in China, which is $10,425.29.3λ is $31,275.88.

### One-Way SA Results at C_*λ*_ and C_3*λ*_

**FIGURE 2:**
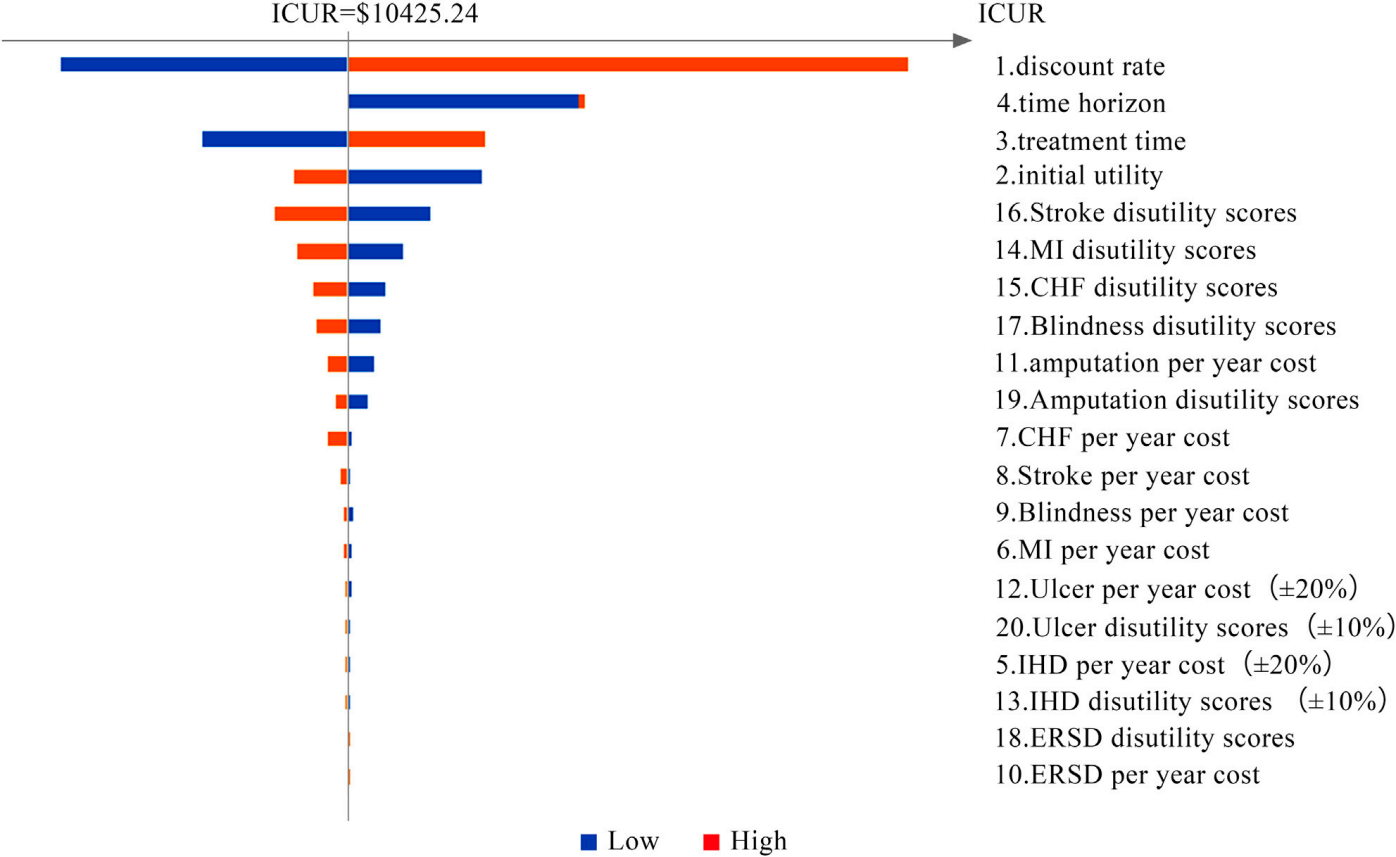
Tornado diagram of the one-way SA (at C_*λ*_ = $708.11).

**FIGURE 3:**
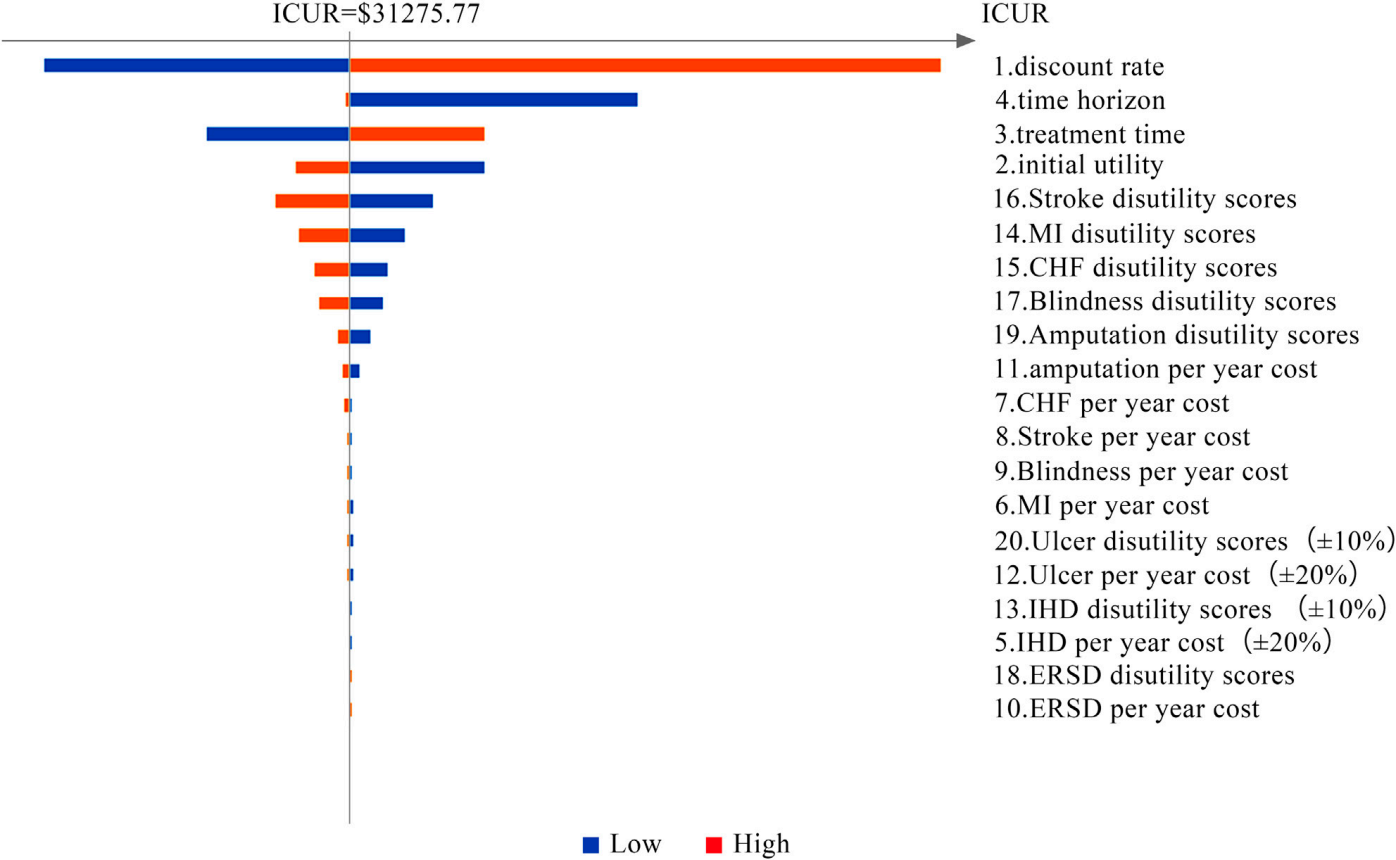
Tornado diagram of the one-way SA (at C_3*λ*_ = $1,007.18).

Tornado diagrams are presented in Figures 2 and 3 to measure the relationship between the ICUR and input parameters at C_*λ*_ and C_3*λ*_, respectively. Twenty potential parameters were assessed in the one-way SA, among which the discount rate has the greatest impact on the results of the cost-utility analysis. Therefore, in the next part, the discount rate was preset at 8%.

### Results for Searching for $C_{\lambda }^{\prime }$ and $C_{3\lambda }^{\prime }$ Using Binary Search at a Discount Rate of 8%

Detailed procedures and results for searching for $C_{\lambda }^{\prime }$ and $C_{3\lambda }^{\prime }$ using binary search at the discount rate of 8% are exhibited in Supplementary Tables S3 and S4. From Table 7, at a discount rate of 8%, when the annual cost of semaglutide was set at $877.43 and $667.04, the ICUR of the OW semaglutide group vs. the OD empagliflozin group was approximately 3*λ* and *λ*, respectively. This means that the cost-utility analysis conclusion of “the incremental cost of OW semaglutide vs. OD empagliflozin appears to be acceptable” was robust to one-way SA when the annual cost of semaglutide was $877.43. In the same way, the cost-utility analysis conclusion of “the incremental cost of OW semaglutide vs. OD empagliflozin appears to be definitely worthwhile” was robust to one-way SA when the annual cost of semaglutide was $667.04. In the next part, PSA and scenario analysis were performed to verify the conclusion.

**TABLE 7:** Results for $C_{\lambda }^{\prime }$ and $C_{3\lambda }^{\prime }$ at the discount rate of 8%.

C_SEMA_	Group	QALY	∆QALY	Total cost	∆Cost	ICUR	Relationship with *λ*
At $C_{3\lambda }^{\prime }$ = $877.43							
	SEMA	8.68	0.06	21,080.25	1,778.90	31,275.85	≈3*λ*
	EMPA	8.62		19,301.35			
At $C_{\lambda }^{\prime }$= $667.04							
	SEMA	8.68	0.06	19,894.31	592.97	10,425.25	≈*λ*
	EMPA	8.62		19,301.35			

$C_{3\lambda }^{\prime }$: annual cost of semaglutide when ICUR was nearly approach to 3*λ* at the discount rate of 8%.

$C_{\lambda }^{\prime }$: annual cost of semaglutide when ICUR was nearly approach to *λ* at the discount rate of 8%.

### Results of Probabilistic Sensitivity Analysis and Scenario Analysis

In the scenario analysis, a 20% reduction in the annual cost of empagliflozin was applied on the basis of the OOP price. From Table 8, when the annual cost of semaglutide at $877.43 and the annual cost of empagliflozin was reduced by 20%, the ICUR was lower than 3*λ*, which means that OW semaglutide was more cost effective than OD empagliflozin. Analogically, the OW semaglutide group was much more cost effective than the OD empagliflozin group, as the ICUR was less than *λ*, when the annual cost of semaglutide was $667.04 and the annual cost of empagliflozin was reduced by 20%. In summary, the results of scenario analysis demonstrated that the conclusion was reliable.

**TABLE 8:** The results of scenario analysis on C_EMPA_.

Group	QALY	∆QALY	Total cost, $	∆cost, $	ICUR, $	
C_SEMA_ at $877.43						
SEMA	11.11	0.09	26,455.02	2,094.07	22,231.90	<3*λ*
EMPA	11.01		24,360.95			
C_SEMA_ at $877.43, C_EMPA_ down 20%						
SEMA	11.11	0.09	26,455.02	2,833.94	30,086.74	<3*λ*
EMPA	11.01		23,621.08			
C_SEMA_ at $667.04						
SEMA	11.11	0.09	25,073.29	712.34	7,562.65	<*λ*
EMPA	11.01		24,360.95			
C_SEMA_ at $667.04, C_EMPA_ down 20%						
SEMA	11.11	0.09	25,073.29	1,452.21	15,417.49	>*λ*
EMPA	11.01		23,621.08			

C_SEMA_: annual cost of semaglutide; C_EMPA_: annual cost of empagliflozin.

**FIGURE 4:**
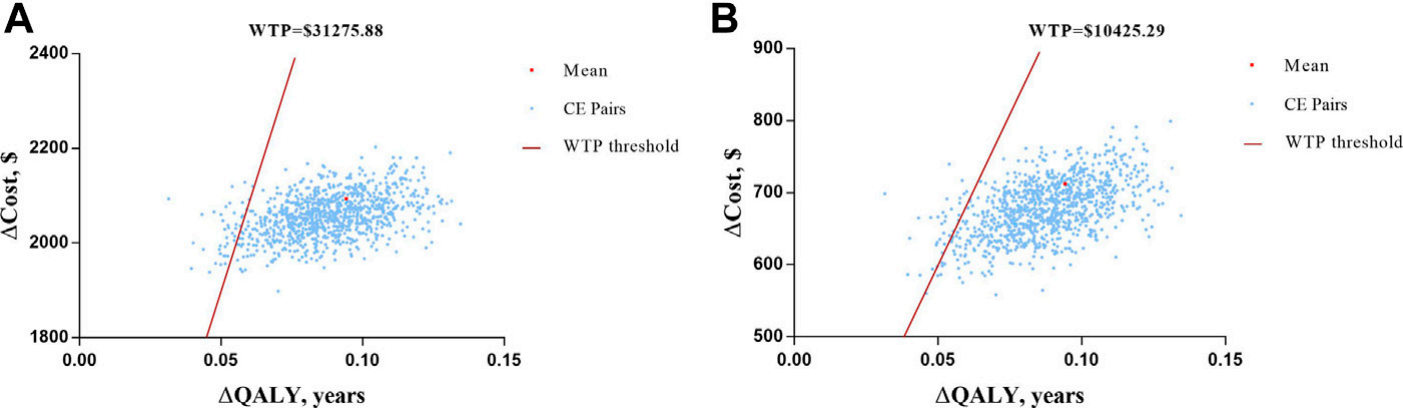
Scatter plots of ICUR for the treatment with semaglutide vs. empagliflozin. **(A)** At the assumption of annual cost of semaglutide of $667.04 with a WTP threshold value of $10,425.29. **(B)** At the assumption of annual cost of semaglutide of $877.43 with a WTP threshold value of $31,275.88.

In PSA, over 1,000 iterations of the Monte Carlo method were simulated with a fixed distribution. Scatter plots of ICUR for the treatment with semaglutide vs. empagliflozin are depicted in Figure 4. All the CE pairs were located in the first quadrant, which means that OW semaglutide conferred more QALY benefits and higher total costs. However, most of the simulations were below the WTP line, which means that OW semaglutide was superior to or more economical with high probability than OD empagliflozin. Compared with OD empagliflozin, OW semaglutide had a 93.4% probability of being very cost effective at an annual cost of semaglutide of $667.04 and a 91.1% probability of being cost effective at an annual cost of semaglutide of $877.43. Therefore, the cost-utility analysis results were robust to PSA.

## Discussion

The risk of cardiovascular diseases is 2- to 4-fold higher in patients with T2D than in nondiabetic individuals, and cardiovascular disease remains the leading cause of death (Kenny and Abel, 2019; De Marco et al., 1999). Therefore, reducing the incidence of cardiovascular complications has become the main goal of diabetes treatment. Thus far, GLP-1 RAs and SGLT-2 inhibitors have shown significant clinical benefits for major cardiovascular events (MACEs) (Bethel et al., 2018; Palmer et al., 2021; Kosiborod et al., 2018; Tsapas et al., 2020). A previous meta-analysis found similar efficacy and safety profiles of GLP-1 RAs and SGLT-2 inhibitors with regard to cardiovascular events (Fei et al., 2019). In a similar study, Zelniker et al. (2019) indicated that GLP-1 RAs and SGLT-2 inhibitors reduce the risk of MACEs to an analogous degree in patients with established atherosclerotic cardiovascular disease (ASCVD). Empagliflozin, a highly selective SGLT-2 inhibitor, is the world’s first hypoglycemic drug that has been confirmed by a large cardiovascular outcome study (EMPA-REG OUTCOME^®^) to reduce the risk of cardiovascular death (Kenny and Abel, 2019). This oral hypoglycemic drug was approved by the National Medical Products Administration (NMPA) on September 26, 2017, to be marketed in China. Among the GLP-1 RAs, semaglutide demonstrated a comparative advantage in reducing glycosylated hemoglobin and the incidence of hypoglycemia events (Ahmed et al., 2018). Focusing on semaglutide, Novo Nordisk developed Ozempic (semaglutide injection) and Rybelsus (oral semaglutide), both of which global blockbuster innovative drugs that are expected to enter the Chinese market. Ozempic is a semaglutide injection that is injected once a week, and Rybelsus is Novo Nordisk’s oral form of semaglutide that was approved by the FDA in September 2019. As the world’s first oral version of a GLP-1 RA, Rybelsus is expected to subvert the current GLP-1 receptor agonist market with better patient compliance experience and efficacy advantages. In general, semaglutide and empagliflozin have both been described to possess favorable cardiovascular protective effects and reduce renal events (Bluhmki et al., 2015; Ipp et al., 2017). Moreover, they have both shown significant advantages in terms of efficacy and safety compared to the same types of drugs. In reviewing the literature, OD empagliflozin was used as a counterpart for OW semaglutide pricing via cost-utility analysis.

To date, this study is the first to compare the long-term cost-effectiveness of OW semaglutide and OD empagliflozin for T2D patients by using the UKPDS OM2 and exploring the appropriate price of OW semaglutide in China based on a binary search. The pharmacokinetics, safety and tolerability of OW subcutaneous semaglutide were assessed by a clinical pharmacology trial conducted in China (Shi et al., 2021). The trial pointed out that it was not necessary to make dose adjustments with the treatment of OW semaglutide in Chinese T2D patients. Meanwhile, several studies have focused on the cost-effectiveness of semaglutide in other countries. Hunt et al. (2019) found that oral semaglutide 14 mg was associated with improved clinical outcomes and a lower cost. Furthermore, a long-term cost-effectiveness analysis demonstrated that oral semaglutide was more cost effective than empagliflozin, sitagliptin, and liraglutide for patients with T2D in the United Kingdom (Bain et al., 2020). Hansen et al. (2020) identified that when two glycaemic lowering goals, HbA1c ≤ 6.5% and HbA1c < 7.0%, are reached, the cost of other subcutaneous injections of GLP-1 receptor agonists is higher than that of semaglutide. Johansen et al. (2020) indicated that compared with once-daily liraglutide 1.2 mg, the life expectancy with once-weekly semaglutide 1 mg increased by 0.21 years, and the quality-adjusted life expectancy increased by 0.3 years. In terms of long-term or short-term cost-effectiveness, the choice of semaglutide to treat T2D is significantly better than that of other antihyperglycaemic drugs.

Our study found that OW semaglutide appears to be superior to OD empagliflozin, with higher QALYs and lower total costs at the initial assumption that the annual cost of semaglutide is equal to that of empagliflozin. In this assumption, the annual cost of semaglutide may be lower than the market price, and it is necessary to increase the annual cost of semaglutide to meet market demand. Therefore, a series of assumptions using binary search for the annual cost of semaglutide were input into the UKPDS OM2 to explore the appropriate price for semaglutide. Consequently, the ICUR of OW semaglutide vs. OD empagliflozin was approximately 3*λ* with an annual cost of semaglutide of $1,007.18 and approximately *λ* with an annual cost of semaglutide of $708.11. Subsequently, the ICUR of OW semaglutide vs. OD empagliflozin was approximately 3*λ* and *λ* with $877.43 and $667.04, respectively, adjusted by one-way SA. Ultimately, the cost-utility results with annual costs of $877.43 and $667.04 were robust to PSA and scenario analysis. This means that OW semaglutide appears to be very cost effective and cost effective to OD empagliflozin, with a range of $667.04 and $877.43 for the annual cost of semaglutide for T2D patients in China.

However, there are several potential limitations of this research. First, long-term economic outcomes were simulated by using the UKPDS 82 equations (Hayes et al., 2013), as these are based on data from White Caucasian, Afro-Caribbean and Asian-Indian populations. Therefore, caution should be taken when extrapolating the model results to other populations (such as the Chinese population). Second, this research relied on short-term clinical trial data to make long-term estimations. Nevertheless, this remains one of the basic principles of pharmacoeconomic modeling for decision-making due to the absence of long-term data. Furthermore, one-way SA, PSA and scenario analysis were applied to solve the clinical doubts about the accuracy of this method. Third, clinical efficacy and safety data were integrated from the SUSTAIN 2, SUSTAIN 3, SUSTAIN 8 and PIONEER 2 trials. In the patient cohort, 77% of patients were white and only 14% of patients were Asian. Necessarily, the results of this study should be adjusted to mirror the real efficacy and safety for patients with real-world evidence in China, on account of a lack of long-term real-world studies for T2D patients treated with OW semaglutide or OD empagliflozin in China. Finally, drug pricing is a complicated procedure based on government-guided pricing and market-adjusted pricing. Physicians and economists have recently appealed to value-based pricing (VBP), which is intended to reduce costs while motivating inspiration for pharmaceutical companies to invent new drugs in the United States and the United Kingdom (Parker-Lue et al., 2015). However, in this study, drug pricing program was explored using pharmacoeconomic methods and provides a reference for semaglutide pricing after being listed in China. Hence, when the conclusion of the study is actually applied to the market decisions, the impacts of other factors need to be considered simultaneously.

These findings provide a value-based range for the annual cost of semaglutide from $667.04 to $877.43. This study demonstrates a framework for exploring value-based pricing for new hypoglycemic agents entering the China marketplace.

## Conclusion

The main goal of the current study was to explore the appropriate price for OW semaglutide in the Chinese market. In conclusion, from the Chinese healthcare provider’s perspective, OW semaglutide appears to be very cost-effective than OD empagliflozin, with an annual cost of semaglutide of $667.04. Analogically, OW semaglutide appears to be more cost-effective than OD empagliflozin, with an annual cost of semaglutide of $877.43. The economic results were robust to one-way SA, PSA and scenario analysis. Therefore, the annual cost of semaglutide appears to be appropriate between $667.04 and $877.43 for T2D patients in China.

## Data Availability

The original contributions presented in the study are included in the article/Supplementary Material, further inquiries can be directed to the corresponding author.
