# Supplementary material for: Exploring the Appropriate Price of Semaglutide for Type 2 Diabetes Patients Based on Cost-Utility Analysis in China
Source: Front Pharmacol. 2021 Jun 10;12:701446. doi: 10.3389/fphar.2021.701446 (PMC8220214; doi:10.3389/fphar.2021.701446)
Supplement: Supplementary file 1 [file DataSheet1.docx]

# Exploring the Appropriate Price of Semaglutide for Type 2 Diabetes Patients Based on Cost-utility Analysis in China

**Shanshan Hu^1, 2†^, Xiaorong Su^1†^, Xun Deng^1, 3^, Yong Wang^1, 3*^**

﻿**^†^**These authors have contributed equally to this work and share first authorship

1. Department of Pharmacy, Zhujiang Hospital, Southern Medical University, Guangzhou 510282, Guangdong, China

2. Department of Clinical Pharmacy, Shanghai General Hospital, Shanghai Jiao Tong University School of Medicine, Shanghai 200080, China

3. Laboratory of Research of New Chinese Medicine, Zhujiang Hospital, Southern Medical University, Guangzhou 510282, Guangdong, China

*** Correspondence:**Corresponding Author
wy43555@smu.edu.cn

Supplementary Material

**Table S1 Tests for a suit price for SEMA using binary search**

|  | annual cost of SEMA, $ | definition, times |
| --- | --- | --- |
| initial | 558.20 | 1 |
| test 1 | 1116.40 | 2 |
| test 2 | 2232.80 | 4 |
| test 3 | 3349.20 | 6 |
| test 4 | 837.30 | 1.5 |
| test 5 | 976.85 | 1.75 |
| test 6 | 1046.63 | 1.875 |
| test 7 | 1011.74 | 1.8125 |
| test 8 | 994.29 | 1.78125 |
| test 9 | 1003.02 | 1.796875 |
| test 10 | 1007.38 | 1.8046875 |
| test 11 | 1005.20 | 1.80078125 |
| test 12 | 1006.29 | 1.802734375 |
| test 13 | 1006.83 | 1.803710938 |
| test 14 | 1007.10 | 1.804199219 |
| test 15 | 1007.24 | 1.804443359 |
| test 16 | 1007.17 | 1.804321289 |
| test 17 | 1007.21 | 1.804382324 |
| test 18 | 1007.19 | 1.804351807 |
| test 19 | 1007.18 | 1.804336548 |
| test 20 | 1007.18 | 1.804328918 |
| test 21 | 697.75 | 1.25 |
| test 22 | 767.53 | 1.375 |
| test 23 | 732.64 | 1.3125 |
| test 24 | 715.19 | 1.28125 |
| test 25 | 706.47 | 1.265625 |
| test 26 | 710.83 | 1.2734375 |
| test 27 | 708.65 | 1.26953125 |
| test 28 | 707.56 | 1.267578125 |
| test 29 | 708.11 | 1.268554688 |
| test 30 | 708.38 | 1.269042969 |
| test 31 | 708.24 | 1.268798828 |
| test 32 | 708.18 | 1.268676758 |
| test 33 | 708.14 | 1.268615723 |
| test 34 | 708.12 | 1.268585205 |
| test 35 | 708.12 | 1.268569946 |
| test 36 | 708.11 | 1.268562317 |
| test 37 | 708.11 | 1.268558502 |
| test 38 | 708.11 | 1.268556595 |

**Table S2 Outputs for cost-utility analysis testing for a suit price for SEMA**

|  | QALY | ∆QALY | total cost | ∆cost | ICUR |  |
| --- | --- | --- | --- | --- | --- | --- |
| initial |  |  |  |  |  |  |
| SEMA | 11.1068 | 0.0942 | 24358.4878 | -2.4602 | dominance | - |
| EMPA | 11.0216 |  | 24360.9480 |  |  |  |
| test 1 2times |  |  |  |  |  |  |
| SEMA | 11.1068 | 0.0942 | 28024.4484 | 3663.5004 | 38890.6624 | >3λ |
| EMPA | 11.0216 |  | 24360.9480 |  |  |  |
| test 4 1.5times |  |  |  |  |  |  |
| SEMA | 11.1068 | 0.0942 | 26191.4681 | 1830.5201 | 19432.2728 | <3λ |
| EMPA | 11.0216 |  | 24360.9480 |  |  |  |
| test 5 1.75times |  |  |  |  |  |  |
| SEMA | 11.1068 | 0.0942 | 27107.9582 | 2747.0102 | 29161.4671 | <3λ |
| EMPA | 11.0216 |  | 24360.9480 |  |  |  |
| test 6 1.875times |  |  |  |  |  |  |
| SEMA | 11.1068 | 0.0942 | 27566.2033 | 3205.2553 | 34026.0648 | >3λ |
| EMPA | 11.0216 |  | 24360.9480 |  |  |  |
| test 7 1.8125times |  |  |  |  |  |  |
| SEMA | 11.1068 | 0.0942 | 27337.0808 | 2976.1328 | 31593.76645 | >3λ |
| EMPA | 11.0216 |  | 24360.9480 |  |  |  |
| test 8 1.78125times |  |  |  |  |  |  |
| SEMA | 11.1068 | 0.0942 | 27222.5195 | 2861.5715 | 30377.61677 | <3λ |
| EMPA | 11.0216 |  | 24360.9480 |  |  |  |
| test 9 1.796875times |  |  |  |  |  |  |
| SEMA | 11.1068 | 0.0942 |  | 2918.8521 | 30985.69108 | <3λ |
| EMPA | 11.0216 |  | 24360.9480 |  |  |  |
| test 10 1.8046875times |  |  |  |  |  |  |
| SEMA | 11.1068 | 0.0942 | 27308.4405 | 2947.4924 | 31289.72824 | >3λ |
| EMPA | 11.0216 |  | 24360.9480 |  |  |  |
| test 11 1.80078125times |  |  |  |  |  |  |
| SEMA | 11.1068 | 0.0942 | 27294.1203 | 2933.1223 | 31137.17941 | <3λ |
| EMPA | 11.0216 |  | 24360.9480 |  |  |  |
| test 12 1.802734375times |  |  |  |  |  |  |
| SEMA | 11.1068 | 0.0942 | 27301.2804 | 2940.3324 | 31213.71975 | <3λ |
| EMPA | 11.0216 |  | 24360.9480 |  |  |  |
| test 13 1.8037109375times |  |  |  |  |  |  |
| SEMA | 11.1068 | 0.0942 | 27304.8604 | 2943.9124 | 31251.72399 | <3λ |
| EMPA | 11.0216 |  | 24360.9480 |  |  |  |
| test 14 1.80419921875times |  |  |  |  |  |  |
| SEMA | 11.1068 | 0.0942 | 27306.6504 | 2945.7024 | 31270.72611 | <3λ |
| EMPA | 11.0216 |  | 24360.9480 |  |  |  |
| test 15 1.804443359375times |  |  |  |  |  |  |
| SEMA | 11.1068 | 0.0942 | 27307.5454 | 2946.5974 | 31280.22718 | >3λ |
| EMPA | 11.0216 |  | 24360.9480 |  |  |  |
| test 16 1.8043212890625times |  |  |  |  |  |  |
| SEMA | 11.1068 | 0.0942 | 27307.0979 | 2946.1499 | 31275.47665 | <3λ |
| EMPA | 11.0216 |  | 24360.9480 |  |  |  |
| test 17 1.80438232421875times |  |  |  |  |  |  |
| SEMA | 11.1068 | 0.0942 | 27307.32165 | 2946.37365 | 31277.85191 | >3λ |
| EMPA | 11.0216 |  | 24360.9480 |  |  |  |
| test 18 1.80435180664062times |  |  |  |  |  |  |
| SEMA | 11.1068 | 0.0942 | 27307.20978 | 2946.261775 | 31276.66428 | >3λ |
| EMPA | 11.0216 |  | 24360.9480 |  |  |  |
| test 19 1.80433654785156times |  |  |  |  |  |  |
| SEMA | 11.1068 | 0.0942 | 27307.15384 | 2946.205838 | 31276.07046 | >3λ |
| EMPA | 11.0216 |  | 24360.9480 |  |  |  |
| test 20 1.80432891845703times |  |  |  |  |  |  |
| SEMA | 11.1068 | 0.0942 | 27307.12587 | 2946.177869 | 31275.77355 | <3λ |
| EMPA | 11.0216 |  | 24360.9480 |  |  |  |
| test 21 1.25times |  |  |  |  |  |  |
| SEMA | 11.1068 | 0.0942 | 25274.97795 | 914.02995 | 9703.078025 | <λ |
| EMPA | 11.0216 |  | 24360.9480 |  |  |  |
| test 22 1.375times |  |  |  |  |  |  |
| SEMA | 11.1068 | 0.0942 | 25733.22303 | 1372.275025 | 14567.67541 | >λ |
| EMPA | 11.0216 |  | 24360.9480 |  |  |  |
| test 23 1.3125times |  |  |  |  |  |  |
| SEMA | 11.1068 | 0.0942 | 25504.10049 | 1143.152488 | 12135.37672 | >λ |
| EMPA | 11.0216 |  | 24360.9480 |  |  |  |
| test 24 1.28125times |  |  |  |  |  |  |
| SEMA | 11.1068 | 0.0942 | 25389.53922 | 1028.591219 | 10919.22737 | >λ |
| EMPA | 11.0216 |  | 24360.9480 |  |  |  |
| test 25 1.265625times |  |  |  |  |  |  |
| SEMA | 11.1068 | 0.0942 | 25332.25858 | 971.3105844 | 10311.1527 | <λ |
| EMPA | 11.0216 |  | 24360.9480 |  |  |  |
| test 26 1.2734375times |  |  |  |  |  |  |
| SEMA | 11.1068 | 0.0942 | 25360.8989 | 999.9509016 | 10615.19004 | >λ |
| EMPA | 11.0216 |  | 24360.9480 |  |  |  |
| test 27 1.26953125times |  |  |  |  |  |  |
| SEMA | 11.1068 | 0.0942 | 25346.57874 | 985.630743 | 10463.17137 | >λ |
| EMPA | 11.0216 |  | 24360.9480 |  |  |  |
| test 28 1.267578125times |  |  |  |  |  |  |
| SEMA | 11.1068 | 0.0942 | 25339.41866 | 978.4706637 | 10387.16203 | <λ |
| EMPA | 11.0216 |  | 24360.9480 |  |  |  |
| test 29 1.2685546875times |  |  |  |  |  |  |
| SEMA | 11.1068 | 0.0942 | 25342.9987 | 982.0507033 | 10425.1667 | <λ |
| EMPA | 11.0216 |  | 24360.9480 |  |  |  |
| test 30 1.26904296875times |  |  |  |  |  |  |
| SEMA | 11.1068 | 0.0942 | 25344.78872 | 983.8407231 | 10444.16903 | >λ |
| EMPA | 11.0216 |  | 24360.9480 |  |  |  |
| test 31 1.268798828125times |  |  |  |  |  |  |
| SEMA | 11.1068 | 0.0942 | 25343.89371 | 982.9457132 | 10434.66787 | >λ |
| EMPA | 11.0216 |  | 24360.9480 |  |  |  |
| test 32 1.2686767578125times |  |  |  |  |  |  |
| SEMA | 11.1068 | 0.0942 | 25343.44621 | 982.4982083 | 10429.91728 | >λ |
| EMPA | 11.0216 |  | 24360.9480 |  |  |  |
| test 33 1.26861572265625times |  |  |  |  |  |  |
| SEMA | 11.1068 | 0.0942 | 25343.22246 | 982.2744558 | 10427.54199 | >λ |
| EMPA | 11.0216 |  | 24360.9480 |  |  |  |
| test 34 1.26858520507812times |  |  |  |  |  |  |
| SEMA | 11.1068 | 0.0942 | 25343.11058 | 982.1625796 | 10426.35435 | >λ |
| EMPA | 11.0216 |  | 24360.9480 |  |  |  |
| test 35 1.26856994628906times |  |  |  |  |  |  |
| SEMA | 11.1068 | 0.0942 | 25343.05464 | 982.1066414 | 10425.76052 | >λ |
| EMPA | 11.0216 |  | 24360.9480 |  |  |  |
| test 36 1.26856231689453times |  |  |  |  |  |  |
| SEMA | 11.1068 | 0.0942 | 25343.02667 | 982.0786724 | 10425.46361 | >λ |
| EMPA | 11.0216 |  | 24360.9480 |  |  |  |
| test 37 1.26855850219727times |  |  |  |  |  |  |
| SEMA | 11.1068 | 0.0942 | 25343.01269 | 982.0646879 | 10425.31516 | >λ |
| EMPA | 11.0216 |  | 24360.9480 |  |  |  |
| test 38 1.26855659484863times |  |  |  |  |  |  |
| SEMA | 11.1068 | 0.0942 | 25343.0057 | 982.0576956 | 10425.24093 | <λ |
| EMPA | 11.0216 |  | 24360.9480 |  |  |  |

**Table S3 Results for searching for C’_3λ_ using binary search at the discount rate of 8%**

| C_SEMA_, $ | Group | QALY | ∆QALY | total cost | ∆cost | ICUR |  |
| --- | --- | --- | --- | --- | --- | --- | --- |
| 708.11 | SEMA | 8.68 | 0.06 | 20125.83 | 824.49 | 14495.75 | >λ |
|  | EMPA | 8.62 |  | 19301.35 |  |  |  |
| 1007.18 | SEMA | 8.68 | 0.06 | 21811.60 | 2510.25 | 44134.09 | >3λ |
|  | EMPA | 8.62 |  | 19301.35 |  |  |  |
| 857.645 | SEMA | 8.68 | 0.06 | 20968.71 | 1667.37 | 29314.92 | <3λ |
|  | EMPA | 8.62 |  | 19301.35 |  |  |  |
| 932.4125 | SEMA | 8.68 | 0.06 | 21390.16 | 2088.81 | 36724.50 | >3λ |
|  | EMPA | 8.62 |  | 19301.35 |  |  |  |
| 895.02875 | SEMA | 8.68 | 0.06 | 21179.43 | 1878.09 | 33019.71 | >3λ |
|  | EMPA | 8.62 |  | 19301.35 |  |  |  |
| 876.336875 | SEMA | 8.68 | 0.06 | 21074.07 | 1772.73 | 31167.32 | <3λ |
|  | EMPA | 8.62 |  | 19301.35 |  |  |  |
| 885.6828125 | SEMA | 8.68 | 0.06 | 21126.75 | 1825.41 | 32093.51 | >3λ |
|  | EMPA | 8.62 |  | 19301.35 |  |  |  |
| 881.0098438 | SEMA | 8.68 | 0.06 | 21100.41 | 1799.07 | 31630.42 | >3λ |
|  | EMPA | 8.62 |  | 19301.35 |  |  |  |
| 878.6733594 | SEMA | 8.68 | 0.06 | 21087.24 | 1785.90 | 31398.87 | >3λ |
|  | EMPA | 8.62 |  | 19301.35 |  |  |  |
| 877.5051172 | SEMA | 8.68 | 0.06 | 21080.66 | 1779.31 | 31283.09 | >3λ |
|  | EMPA | 8.62 |  | 19301.35 |  |  |  |
| 876.9209961 | SEMA | 8.68 | 0.06 | 21077.37 | 1776.02 | 31225.20 | <3λ |
|  | EMPA | 8.62 |  | 19301.35 |  |  |  |
| 877.2130566 | SEMA | 8.68 | 0.06 | 21079.01 | 1777.67 | 31254.15 | <3λ |
|  | EMPA | 8.62 |  | 19301.35 |  |  |  |
| 877.3590869 | SEMA | 8.68 | 0.06 | 21079.84 | 1778.49 | 31268.62 | <3λ |
|  | EMPA | 8.62 |  | 19301.35 |  |  |  |
| 877.4321021 | SEMA | 8.68 | 0.06 | 21080.25 | 1778.90 | 31275.85 | <3λ |
|  | EMPA | 8.62 |  | 19301.35 |  |  |  |
| 877.4686096 | SEMA | 8.68 | 0.06 | 21080.45 | 1779.11 | 31279.47 | >3λ |
|  | EMPA | 8.62 |  | 19301.35 |  |  |  |

**Table 4 Results for searching for C’_λ_ using binary search at the discount rate of 8%**

| C_SEMA_, $ | Group | QALY | ∆QALY | total cost | ∆cost | ICUR |  |
| --- | --- | --- | --- | --- | --- | --- | --- |
| 708.11 | SEMA | 8.68 | 0.06 | 20125.83 | 824.49 | 14495.75 | >λ |
|  | EMPA | 8.62 |  | 19301.35 |  |  |  |
| 558.20 | SEMA | 8.68 | 0.06 | 19280.84 | -20.51 | -360.58 | <λ |
|  | EMPA | 8.62 |  | 19301.35 |  |  |  |
| 633.155 | SEMA | 8.68 | 0.06 | 19703.33 | 401.99 | 7067.58 | <λ |
|  | EMPA | 8.62 |  | 19301.35 |  |  |  |
| 670.6325 | SEMA | 8.68 | 0.06 | 19914.58 | 613.24 | 10781.67 | >λ |
|  | EMPA | 8.62 |  | 19301.35 |  |  |  |
| 651.89375 | SEMA | 8.68 | 0.06 | 19808.96 | 507.61 | 8924.62 | <λ |
|  | EMPA | 8.62 |  | 19301.35 |  |  |  |
| 661.263125 | SEMA | 8.68 | 0.06 | 19861.77 | 560.43 | 9853.14 | <λ |
|  | EMPA | 8.62 |  | 19301.35 |  |  |  |
| 665.9478125 | SEMA | 8.68 | 0.06 | 19888.18 | 586.83 | 10317.41 | <λ |
|  | EMPA | 8.62 |  | 19301.35 |  |  |  |
| 668.2901563 | SEMA | 8.68 | 0.06 | 19901.38 | 600.03 | 10549.54 | >λ |
|  | EMPA | 8.62 |  | 19301.35 |  |  |  |
| 667.1189844 | SEMA | 8.68 | 0.06 | 19894.78 | 593.43 | 10433.47 | >λ |
|  | EMPA | 8.62 |  | 19301.35 |  |  |  |
| 666.5333984 | SEMA | 8.68 | 0.06 | 19891.48 | 590.13 | 10375.44 | <λ |
|  | EMPA | 8.62 |  | 19301.35 |  |  |  |
| 666.8261914 | SEMA | 8.68 | 0.06 | 19893.13 | 591.78 | 10404.45 | <λ |
|  | EMPA | 8.62 |  | 19301.35 |  |  |  |
| 666.9725879 | SEMA | 8.68 | 0.06 | 19893.95 | 592.61 | 10418.96 | <λ |
|  | EMPA | 8.62 |  | 19301.35 |  |  |  |
| 667.0457861 | SEMA | 8.68 | 0.06 | 19894.37 | 593.02 | 10426.22 | >λ |
|  | EMPA | 8.62 |  | 19301.35 |  |  |  |
| 667.009187 | SEMA | 8.68 | 0.06 | 19894.16 | 592.81 | 10422.59 | <λ |
|  | EMPA | 8.62 |  | 19301.35 |  |  |  |
| 667.0274866 | SEMA | 8.68 | 0.06 | 19894.26 | 592.92 | 10424.40 | <λ |
|  | EMPA | 8.62 |  | 19301.35 |  |  |  |
| 667.0366364 | SEMA | 8.68 | 0.06 | 19894.31 | 592.97 | 10425.31 | >λ |
|  | EMPA | 8.62 |  | 19301.35 |  |  |  |
| 667.0320615 | SEMA | 8.68 | 0.06 | 19894.29 | 592.94 | 10424.86 | <λ |
|  | EMPA | 8.62 |  | 19301.35 |  |  |  |
| 667.0343489 | SEMA | 8.68 | 0.06 | 19894.30 | 592.96 | 10425.08 | <λ |
|  | EMPA | 8.62 |  | 19301.35 |  |  |  |
| 667.0354926 | SEMA | 8.68 | 0.06 | 19894.31 | 592.96 | 10425.20 | <λ |
|  | EMPA | 8.62 |  | 19301.35 |  |  |  |
| 667.0360645 | SEMA | 8.68 | 0.06 | 19894.31 | 592.97 | 10425.25 | <λ |
|  | EMPA | 8.62 |  | 19301.35 |  |  |  |
